# Subjective cognitive, psychiatric, and fatigue symptoms two years after COVID-19: A prospective longitudinal cohort study

**DOI:** 10.1016/j.bbih.2025.100980

**Published:** 2025-03-18

**Authors:** Henriikka Ollila, Marjaana Tiainen, Riikka Pihlaja, Sanna Koskinen, Annamari Tuulio-Henriksson, Viljami Salmela, Laura Hokkanen, Johanna Hästbacka

**Affiliations:** a Perioperative and Intensive Care, Helsinki University Hospital and University of Helsinki, Helsinki, Finland; b Department of Neurology, Helsinki University Hospital and University of Helsinki, Helsinki, Finland; c Department of Psychology, Faculty of Medicine, University of Helsinki, Helsinki, Finland; d Division of Neuropsychology, HUS Neurocenter, Helsinki University Hospital and University of Helsinki, Helsinki, Finland; e Department of Intensive Care, Tampere University Hospital, Wellbeing Services County of Pirkanmaa, Tampere, Finland; f Tampere University, Faculty of Medicine and Health Technology, Tampere, Finland

**Keywords:** COVID-19, Subjective cognition, Depression, Anxiety, Post-traumatic stress, Fatigue, Long-term outcome

## Abstract

**Introduction:**

COVID-19 survivors may present with cognitive and psychiatric symptoms long after the acute phase of SARS-CoV-2 infection.

**Objectives:**

To determine subjective cognitive, psychiatric, and fatigue symptoms two years after COVID-19, and their change from six months to two years.

**Methods:**

We assessed three COVID-19 patient groups of different acute disease severity (ICU-treated, ward-treated, home-isolated) concerning subjective cognitive functioning (AB Neuropsychological Assessment Schedule), anxiety (Generalised Anxiety Disorder 7), depression (Patient Health Questionnaire 9), post-traumatic stress (Impact of Event Scale 6), and fatigue (Multidimensional Fatigue Inventory) with a mailed questionnaire approximately two years after acute COVID-19. We compared the results with those obtained six months after the acute disease. We studied whether any change emerged in the scores of symptomatic patients between six- and 24-month follow-ups.

**Results:**

Two years post-COVID-19, 58 ICU-treated, 35 ward-treated, and 28 home-isolated patients responded to the questionnaire. Subjective cognitive symptoms and fatigue emerged as the most common problems occurring in 30.6 and 35.5% of patients, respectively. In patients with clinically significant symptoms at six months, symptom scores for depression, anxiety, and post-traumatic stress decreased at two years.

**Conclusions:**

Two years after COVID-19, particularly self-reported cognitive symptoms and fatigue remained clinically significant, but also some recovery was evident in depression, anxiety, and post-traumatic stress.

## Highlights

• Two years post-COVID-19, patients suffer from subjective cognitive issues and fatigue.
• Over two years, psychiatric symptom scores in symptomatic COVID-19 patients improved.
• COVID-19 patients symptomatic at 2 years, had more functional disability at 3 months.

## 1 Introduction

After intensive care unit (ICU) treatment, patients can have various physical, cognitive, and psychiatric symptoms for years (Needham et al., 2012). Since the start of the COVID-19 pandemic, reports of persistent cognitive (Andronescu et al., 2024; Ceban et al., 2022), neurologic (Premraj et al., 2022), and psychiatric symptoms (Veazie et al., 2022) in COVID-19 patients emerged. Studies have shown mixed results on the association between level of care or COVID-19 severity and psychiatric or cognitive symptoms (Andronescu et al., 2024; Veazie et al., 2022). On the other hand, reports exist indicating that patients experience no more psychiatric symptoms one to six months post-COVID-19 than the general population (Bourmistrova et al., 2022). During the pandemic, mental health problems increased also in the general population, particularly in those with a previous history of mental health disorders (Young et al., 2023). In an extensive retrospective health record study, risks of mood and anxiety disorders in COVID-19 patients returned to baseline 1–2 months after COVID-19; however, risks of cognitive deficit, dementia, and psychotic disorders remained increased for at least two years (Taquet et al., 2022). The pathophysiology behind these neuropsychiatric problems remains unclear, but some hypotheses include persistent inflammation, neurotropism of SARS-CoV-2, static brain injury that occurred in the acute phase (similar to e.g. stroke), progressive neurodegeneration triggered by hypoxaemia, inflammation, and blood-brain barrier disruption in the acute phase, pandemic-associated psychosocial stressors, and mechanism similar to causing neuropsychiatric problems after critical illness (Ali Awan et al., 2021; Frontera and Simon, 2022). Concerning post-COVID-19 fatigue, intracortical GABAergic dysfunction caused by neuroinflammation might play a role (Campos et al., 2022).

Twelve months post-COVID-19, in several studies, hospitalised and non-hospitalised patients suffered from fatigue, anxiety, depression, post-traumatic stress disorder (PTSD), and cognitive problems (Albtoosh et al., 2022; Bek et al., 2023; Fernandez-de-Las-Penas et al., 2022a; Han et al., 2022; Hussain et al., 2024; Li et al., 2023). Risk factors for prolonged symptoms included female sex, older age, fewer years of education, history of chronic disease, life stressors, and more severe acute phase illness (Cavaco et al., 2023; Frontera et al., 2022a; Han et al., 2022; Hussain et al., 2024; Li et al., 2023). In addition, cognitive complaints were associated with symptoms of anxiety, depression, post-traumatic stress, and fatigue, and showed a moderate or no association with objective cognitive functioning (Bek et al., 2023; Cavaco et al., 2023; Godoy-Gonzalez et al., 2023; Miskowiak et al., 2022; Pihlaja et al., 2023). In hospitalised patients, during the first 12 months after acute COVID-19, longitudinal studies concerning cognition and mental health have described recovery, but also persistent and often coexisting symptoms (Bek et al., 2023; Evans et al., 2022; Fernandez-de-Las-Penas et al., 2022a; Frontera et al., 2022b; Huang et al., 2021; Kyzar et al., 2021). The duration of these symptoms is uncertain.

We aimed to determine a) if, after a 24-month follow-up, COVID-19 patients of different acute disease severity show subjective cognitive, psychiatric, and fatigue symptoms and if differences exist between those severity groups, b) whether any change in their symptoms over the follow-up period from six to 24 months occurs, c) if any factors common to patients with impaired recovery emerge, and d) what the functional outcome two years after COVID-19 is. In our previous work, we showed that six months after COVID-19, patients reported more subjective cognitive, depressive, and post-traumatic stress symptoms than non-COVID-19 controls, but no differences existed between the different COVID-19 severity groups (Pihlaja et al., 2023). Thus, we hypothesised that patients show some recovery from six months to two years, but ICU patients have a slower recovery trajectory and hence more symptoms at two years. Another hypothesis was that recovery would be most evident in those with clinically significant subjective cognitive, psychiatric or fatigue symptoms at six months. Based on previous studies, we hypothesised that at two years subjective cognitive, psychiatric, and fatigue symptoms co-exist, and can be partly explained by factors like demographics, comorbidities, acute phase disease severity, and early functional and cognitive outcome.

## 2 Materials and methods

**Fig. 1:**
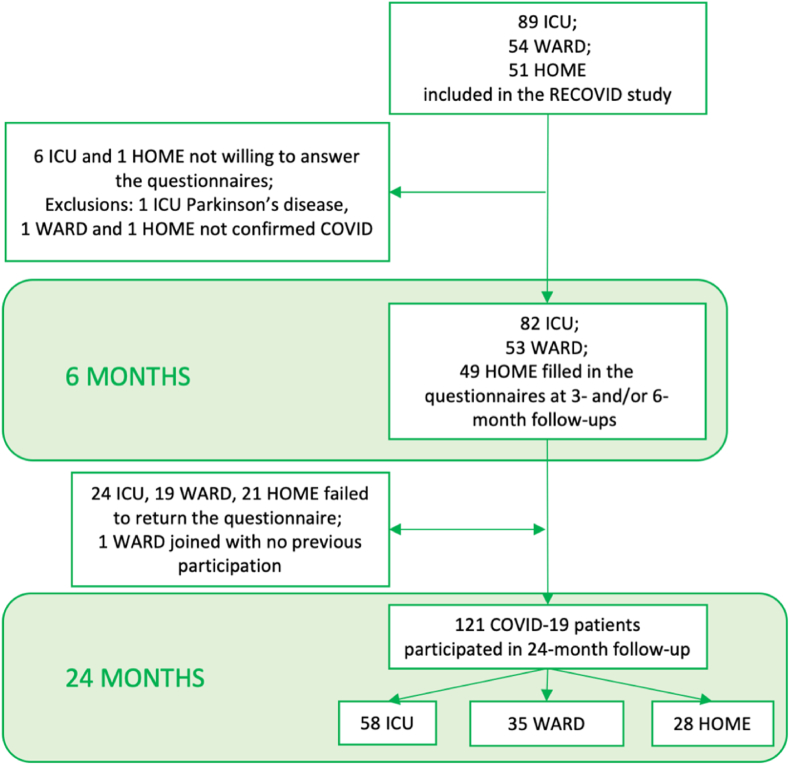
Flow chart of patients included in the analysis.

This is a prospective follow-up study, part of the Recovery after critical COVID-19 infection (RECOVID) study project (ClinicalTrials.gov NCT04864938). The ethics committee of Helsinki University Hospital approved the study protocol (HUS–1949–2020). Written informed consent was obtained from all the patients. We followed the principles of the Declaration of Helsinki. We applied the Strengthening the Reporting of Observational Studies in Epidemiology checklist for cohort studies in reporting the results of this study (von Elm et al., 2007). We have reported the formation of this cohort previously (Ollila et al., 2022). Briefly, adult patients diagnosed with COVID-19 in 2020 were eligible after ICU or ward care or home isolation. This determined the COVID-19 patient groups (ICU, WARD, HOME). Demographics including age, comorbidities, and body mass index (BMI) were collected during or soon after the acute disease phase. Everyone, who formerly consented to the RECOVID study, received follow-up questionnaires. At three months, patients filled out a questionnaire on post-traumatic stress and subjective cognitive symptoms. At six months, the patients underwent a comprehensive neuropsychological assessment. Nine tests were chosen to represent domains of memory, executive functions, and attention as outcome variables. These variables were transformed into Z-scores and, when summed, formed the total cognitive score (Ollila et al., 2022). At the same time point, patients answered questionnaires on subjective cognitive, psychiatric, and fatigue symptoms. At 24 months after the acute phase, patients answered a mailed questionnaire about subjective cognitive, psychiatric, and fatigue symptoms as well as functional and employment status. The final sample of the present study included 121 respondents (Fig. 1).

### 2.1 Assessment

At 24 months, we evaluated subjective cognition with the AB Neuropsychological Assessment Schedule (ABNAS) (Aldenkamp et al., 1995), where a cut-off of >15 points indicates subjective cognitive impairment (Aldenkamp et al., 2002). For depressive symptoms, we employed the Patient Health Questionnaire 9 (PHQ-9) with a cut-off of ≥10 representing moderate or more severe depressive symptoms (Kroenke et al., 2001). Anxiety was evaluated with the Generalised Anxiety Disorder 7 (GAD-7), where a cut-off ≥5 indicates mild or more severe anxiety symptoms (Spitzer et al., 2006). For post-traumatic stress, we applied the Impact of Event Scale 6 (IES-6) with a mean score of >1.75 representing post-traumatic stress disorder (PTSD) symptoms (Hosey et al., 2019). For fatigue, we employed the Multidimensional Fatigue Inventory (MFI) (Smets et al., 1995), where a higher score indicates more fatigue (Hinz et al., 2020). Recently, a cut-off of 60 was introduced for Parkinson's patients (Huether et al., 2023); also a cut-off of >58 has been utilised (Vollrath et al., 2013). We decided to apply the cut-off of ≥60 to be able to describe the prevalence of fatigue in our cohort. In addition, the questionnaire included questions about employment and functional status, out of which an experienced neurologist (M.T.) determined the modified Rankin scale (mRS) (van Swieten et al., 1988), as well as a self-reported number of severe acute respiratory syndrome coronavirus 2 (SARS-CoV-2) infections.

### 2.2 Study outcomes

The main outcomes were a) the presence of clinically significant symptoms in the domains of subjective cognition, depression, anxiety, post-traumatic stress, and fatigue at 24 months in different acute COVID-19 severity groups, b) the change in the prevalence from six to 24 months of clinically significant symptoms, defined by the cut-off limits, as well as the change in the symptom scores, c) factors associated with impaired recovery, and d) functional outcome at 24 months.

### 2.3 Statistics

Descriptive statistics are expressed as median and interquartile range (IQR) or mean and standard deviation (SD) for continuous variables, and as number of subjects and percentage for categorical variables. The Chi^2^ test or Fisher's exact test was used for categorical variables and the non-parametric Kruskal-Wallis test for continuous variables with non-normal distribution. A P-value <0.05 was required for statistical significance. To control for multiple comparisons, we used the false discovery rate (FDR) correction. The FDR-corrected threshold for significance was p = 0.007. For the analyses, we employed Jamovi project® (version 2.3.21.0) and RStudio® (version 2023.06.2 + 561).

If a study subject had not answered a question at six months, we used the three-month follow-up answer, when available (ABNAS and IES-6 occurred at both three and six months). If this was not possible, we used the R mice package for imputation. If only single answers were missing, we imputed those; in the case of the whole domain missing, we imputed the sum or mean variable. We performed imputation also for single missing answers at 24 months. One to five values were imputed for 20 patients (60 imputations in total).

To examine the changes in symptom scores and the frequency of impairment between the two time points, we applied repeated measures ANOVA with Tukey's post-hoc test and McNemar test, respectively. We analysed ABNAS, PHQ-9, GAD-7, IES-6, and MFI at 24 months and total cognitive Z-score at six months for partial correlation with Pearson's test. Repeated measures ANOVA and Pearson's partial correlation were examined as separate analyses and were thus excluded from the FDR correction.

## 3 Results

We will first present our patient cohort; second, describe their level of cognitive, psychiatric, and fatigue symptoms at two years; third, describe the evolution of symptoms in a previously symptomatic cohort; fourth, present the partial correlations between the cognitive, psychiatric, and fatigue symptoms; fifth, describe factors associated with being symptomatic at two years; and sixth, describe the functional outcome.

### 3.1 Patients

At 24 months, out of 185 COVID-19 patients, 58 ICU-treated, 35 ward-treated, and 28 home-isolated COVID-19 patients participated in the follow-up (Fig. 1) which adds up to a total participation of 65.4 %. All, except one WARD subject, had participated in the questionnaire study previously at three and/or six months (Fig. 1). The HOME group was the youngest, and in the ICU group, the proportion of females and number of educational years were lower than in the other groups (Table 1).

**Table 1:** Characteristics and comorbidities of the patients in different acute disease severity groups, and outcomes of subjective cognitive, depressive, anxiety, post-traumatic stress, and fatigue symptoms as well as functional outcome and employment status 24 months after acute COVID-19.

	ICU, n = 58	WARD, n = 35	HOME, n = 28	p
Number of SARS-CoV-2 infections >1, n (%)	10 (17.2)	2 (5.7)	10 (35.7)	0.011^a^
Age^b^, years, median (IQR)	60 (50–67.5)	58 (51.5–62.5)	48.5 (43–58.3)	**0.003**
Sex, female, n (%)	23 (39.7)	25 (71.4)	20 (71.4)	**0.002**
Education years, median (IQR)	14 (12–15)	15 (12.3–17)	15 (15–17)	**0.001**

*Acute phase parameters*				
Hospital LOS, days, median (IQR)	21.5 (16.3–26.8)	9 (5–12)	0	**< 0.001**
ICU LOS, days, median (IQR)	12.5 (6–17.8)	–	–	

*Comorbidities*				
Arterial hypertension, n (%)	30 (51.7)	9 (25.7)	7 (25)	0.012^a^
Heart disease, n (%)	9 (15.5)	1 (2.9)	3 (10.7)	0.183
Diabetes, n (%)	13 (22.4)	3 (8.6)	2 (7.1)	0.099
Asthma, n (%)	6 (10.3)	11 (31.4)	3 (10.7)	0.030
Neurological or psychiatric condition, n (%)	3 (5.2)	4 (11.4)	1 (3.6)	0.450
BMI kg/m^2^, median (IQR) n = 104	29.5 (26.8–34), n = 58	29.0 (25.1–33.6), n = 26	25.3 (23.3–27.8), n = 20	**0.006**

*Subjective cognition*				
ABNAS, median (IQR)	11 (5–19.8)	7 (2.5–25.5)	9.5 (1.8–16.8)	0.702
ABNAS >15, n (%)	17 (29.3)	12 (34.3)	8 (28.6)	0.851

*Depressive symptoms*				
PHQ-9, median (IQR)	3 (1–5.8)	3 (0.5–7)	2.5 (0–6.3)	0.895
PHQ-9 ≥ 10, n (%)	6 (10.3)	6 (17.1)	3 (10.7)	0.637

*Anxiety symptoms*				
GAD-7, median (IQR)	1 (0–5)	3 (0–5)	2 (0–4)	0.868
GAD-7 ≥ 5, n (%)	17 (29.3)	11 (31.4)	5 (17.9)	0.432

*Post-traumatic stress symptoms*				
IES-6, median (IQR)	0.5 (0.2–0.8)	0.5 (0–0.9)	0.2 (0–0.5)	0.036^a^
IES-6 > 1.75, n (%)	6 (10.3)	5 (14.3)	1 (3.6)	0.382

*Fatigue*				
MFI, median (IQR)	48 (38–66.8)	48 (41–60.5)	56 (37.8–62.3)	0.988
MFI ≥60, n (%)	21 (36.2)	9 (25.7)	13 (46.4)	0.230

*Functional outcome*				
mRS, median (IQR)	1 (0–1)	1 (0–1)	0 (0–1)	0.724

*Employment status*				
Working full-time, n (%)	31 (53.4)	19 (54.3)	20 (71.4)	0.252
Working full-time, if in working life before COVID-19, n (%)	31/40 (77.5)	17/25 (68)	18/21 (85.7)	0.362
Old age pension, if in working life before COVID-19, n (%)	6/40 (15)	3/25 (12)	1/21 (4.8)	0.494
Old age pension, n (%)	21 (36.2)	10 (28.6)	4 (14.3)	0.11
On sick leave or disability pension, n (%)	2 (3.4)	2 (5.7)	2 (7.1)	0.652

*SARS-CoV-2* Severe acute respiratory syndrome coronavirus 2; *IQR* interquartile range; *LOS* length of stay; *BMI* Body mass index; *ABNAS* AB Neuropsychological Assessment Schedule; *PHQ-9* Patient Health Questionnaire 9; *GAD-7* Generalised Anxiety Disorder 7; *IES-6* Impact of Event Scale 6; *MFI* Multidimensional Fatigue Inventory; *mRS* modified Rankin Scale. Statistically significant p-values in bold.

a non-significant after FDR correction.

b age when fallen ill.

ICU patients answered the questionnaire a median of 778 days (IQR 746–817) and WARD patients a median of 790 days (IQR 768–819) after hospital discharge (approximately 26 months). HOME patients answered a median of 817 days (IQR 803–844) after the positive test result (approximately 27 months).

When comparing those who failed to answer the follow-up questionnaire to those who returned the questionnaire at 24 months, no significant differences emerged in terms of years of education (median 15 years, IQR 12–17, in both groups, p = 0.954), mRS at three months (median 1, IQR 1–2 in both groups, p = 0.245), proportion of females (56.2 vs 56.3 %, p = 0.995), or acute disease severity group (p = 0.284). However, those who were lost to follow-up were younger (median age 48.5, IQR 38.5–59.0) than those who participated (median age 57, IQR 48–64, p < 0.001). None of the patients had died during the follow-up period.

### 3.2 Subjective cognitive, psychiatric and fatigue outcome

The symptom scores for subjective cognition, depression, anxiety, and post-traumatic stress symptoms, and fatigue at 24 months are presented in Table 1. Almost one-third (30.6%) of all COVID-19 patients exceeded the cut-off for significant symptoms in subjective cognitive functioning, more than one-third (35.5%) in fatigue, 27.3% in anxiety, 12.4% in depression, and 9.9% in post-traumatic stress. No differences in either the median scores or the frequencies of clinically significant symptoms in these domains existed between the different severity groups (Table 1).

### 3.3 Evolution of symptoms over the follow-up period

**Fig. 2:**
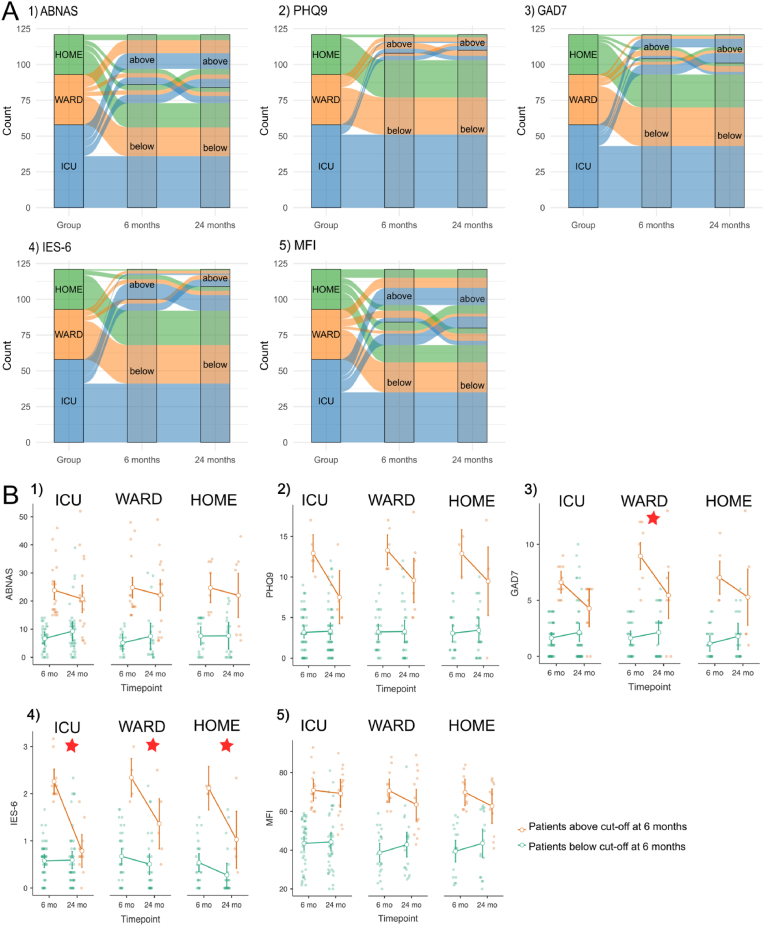
**A)** Alluvial diagrams showing the change over time in the number of patients exceeding cut-off limits for 1) subjective cognition 2) depression, 3) anxiety, 4) post-traumatic stress, and 5) fatigue. Proportions of patients exceeding the cut-off limits were statistically not different between the two time points (McNemar test for paired samples). **B)** Change in mean symptom scores from six to 24 months in different COVID-19 severity groups for 1) subjective cognition, 2) depression, 3) anxiety, 4) post-traumatic stress, and 5) fatigue. Green dots represent patients with scores below cut-off and orange dots represent those with scores above cut-off at six months. For subjective cognition and fatigue, no significant change over time existed (1 and 5). For depressive, anxiety and post-traumatic stress symptoms, patients with scores above cut-off at six months, had significantly lower scores at 24 months (2, 3 and 4). Red stars ★ denote significant changes in group-wise comparisons over the follow-up period. Error bars represent 95% confidence intervals. *ABNAS* AB Neuropsychological Assessment Schedule; *PHQ9* Patient Health Questionnaire 9; *GAD7* Generalised Anxiety Disorder 7; *IES-6* Impact of Event Scale 6; *MFI* Multidimensional Fatigue Inventory. (For interpretation of the references to colour in this figure legend, the reader is referred to the Web version of this article.)

No differences in proportions of patients exceeding cut-off levels for each domain separately at six months compared to 24 months existed; however, there was a trend towards a smaller proportion exceeding the cut-off for post-traumatic stress and a trend towards a greater proportion exceeding the cut-off for anxiety (Fig. 2A and Table S1 in supplementary material). In PHQ-9 for depression, GAD-7 for anxiety, and IES-6 for post-traumatic stress, of those who exceeded the cut-off level at 24 months, more than 50 % were below the cut-off at six months; in ABNAS for subjective cognition and MFI for fatigue that proportion was approximately one in three (Fig. 2A and Table S1).

COVID-19 patients with clinically significant symptoms (i.e. score above cut-off) at six months had lower symptom scores at 24 months in depression (p < 0.001), anxiety (p < 0.001), and post-traumatic stress (p < 0.001); for the asymptomatic patients, no significant change in symptom scores occurred (Fig. 2B and Table S2 in the supplementary material). For subjective cognition and fatigue, no improvement or deterioration over the follow-up occurred (Fig. 2B and Table S2). For anxiety symptoms, the WARD group with clinically significant symptoms at six months had a significantly lower score at 24 months (p = 0.024) (Fig. 2B and Table S2). For post-traumatic stress, ICU, WARD, and HOME groups with clinically significant symptoms at six months had significantly lower scores at 24 months (p < 0.001, p = 0.025, p = 0.030, respectively) (Fig. 2B and Table S2).

### 3.4 Overlap of subjective cognitive, psychiatric, and fatigue symptoms

**Fig. 3:**
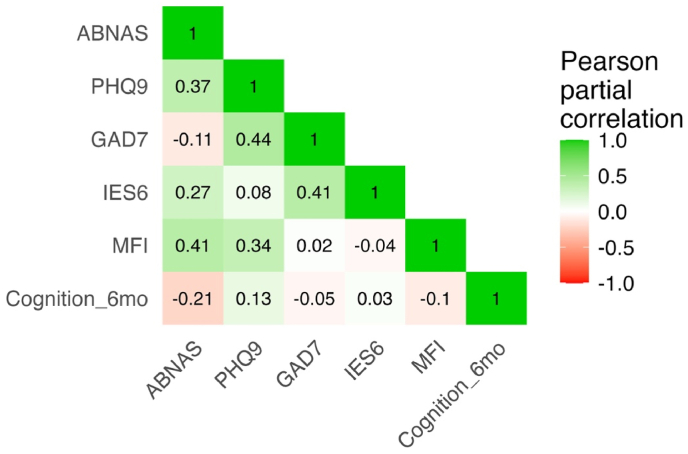
Analysis of partial correlations for ABNAS, PHQ-9, GAD-7, IES-6, and MFI scores at 24 months presented as a heat map. Results are additionally adjusted for age and educational years. The total cognitive Z-score at 6 months (=Cognition_6mo) was included in the analysis. Partial correlations >0.2 are statistically significant (p < 0.05).

After adjusting for age and educational years, a significant positive partial Pearson correlation existed between symptoms of subjective cognition and depression, subjective cognition and post-traumatic stress, subjective cognition and fatigue, depression and anxiety, depression and fatigue, and anxiety and post-traumatic stress (Fig. 3). The analysis included also objective total cognitive score at six months which had a negative correlation with the subjective cognitive score at two years i.e. patients with good performance in complete neuropsychological assessment had lower ABNAS scores. A partial correlation test measures the correlation between the two chosen variables and controls for the impact of the other variables included. A heat map for Pearson correlation, adjusted for age and educational years only, is available in the supplementary material (Fig. S1).

### 3.5 Factors associated with clinically significant cognitive, psychiatric, and fatigue symptoms at 24 months

**Fig. 4:**
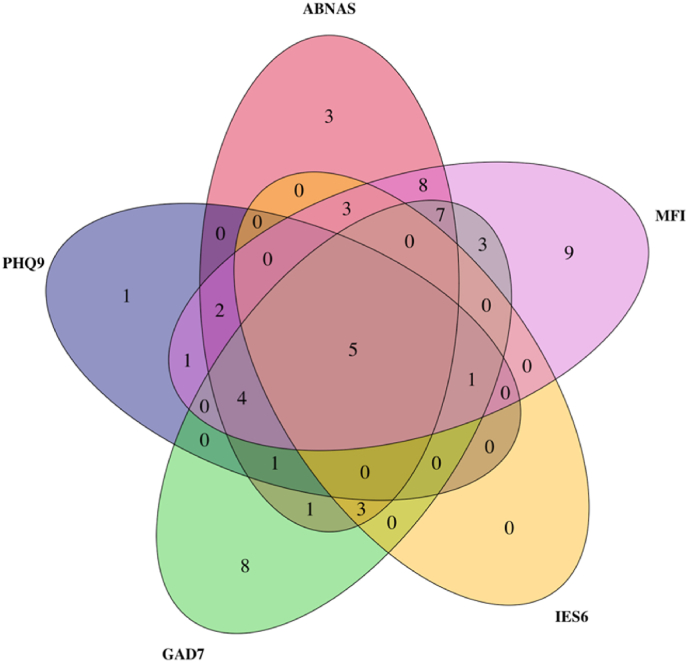
Venn diagram showing the number of patients exceeding the cut-off levels in different domains at 24 months. The total number of patients who scored above any cut-off level was 60; of those, 21 exceeded the cut-off in only one domain. *ABNAS* AB Neuropsychological Assessment Schedule; *PHQ9* Patient Health Questionnaire 9; *GAD7* Generalised Anxiety Disorder 7; *IES-6* Impact of Event Scale 6; *MFI* Multidimensional Fatigue Inventory.

Out of 121 COVID-19 patients, 24 months after COVID-19, 60 (49.6%) experienced clinically significant symptoms (score above cut-off level) in one or more domains of subjective cognition, depression, anxiety, post-traumatic stress or fatigue; 39/121 (32%) exceeded the cut-off in more than one domain (Fig. 4). Five (4%) patients exceeded the cut-off in all five domains (Fig. 4).

No differences in comorbidities or demographic factors existed when COVID-19 patients with a score above the cut-off level in any domain were compared to those who scored below the cut-off (Table 2). Patients exceeding at least one cut-off at 24 months had more disability at three months in terms of mRS (median 2, IQR 1–2 vs median 1, IQR 0–1, p < 0.001) than those below the cut-offs, and a trend towards lower total cognitive Z-score (median −0.71 vs 2.43, p = 0.024) six months after COVID-19 existed (Table 2). The difference in functional outcome persisted at 24 months (mRS median 1, IQR 0–2 vs median 0, IQR 0–1, p < 0.001), but no difference in employment status emerged. Among patients scoring above the cut-off level, 55% were working full-time compared to 60.7% of those with scores below the cut-off, the difference being statistically non-significant (Table 2).

**Table 2:** Characteristics of COVID-19 patients who exceeded the cut-off level in at least one domain (subjective cognition, depression, anxiety, post-traumatic stress, or fatigue) at 24 months compared to those who were below the cut-off level in all domains.

	Below the cut-off level in all domains, n = 61	Above the cut-off level in one or more domains, n = 60	p
Number of SARS-CoV-2 infections >1, n (%)	9 (14.8)	13 (21.7)	0.324
Age, years, median (IQR)	58 (49–64)	56 (47.8–63.3)	0.544
Sex, female, n (%)	31 (50.8)	37 (61.7)	0.229
Education years, median (IQR)	15 (12–17)	15 (12–16)	0.644

*Comorbidities*			
Arterial hypertension, n (%)	20 (32.8)	26 (43.3)	0.232
Heart disease, n (%)	8 (13.1)	5 (8.3)	0.396
Diabetes, n (%)	6 (9.8)	12 (20)	0.116
Asthma, n (%)	8 (13.1)	12 (20)	0.308
Neurological or psychiatric condition, n (%)	2 (3.3)	6 (10)	0.163
Charlson comorbidity index	1.5 (1–3)	2 (1–3)	0.829
BMI kg/m^2^, median (IQR), n = 104	27.7 (24.1–30.7)	29.3 (27.0–34.0)	0.021^a^

*Acute phase parameters*			
Group ICU, n (%)	29 (47.5)	29 (48.3)	0.922
WARD, n (%)	17 (27.9)	18 (30)	
HOME, n (%)	15 (24.6)	13 (21.7)	
Hospital LOS, days, median (IQR)	12 (3–21)	10.5 (3.8–20.5)	0.921
ICU LOS, days, median (IQR), n = 58	10 (5–17)	14 (7–19)	0.346
IMV, n (%)	18 (29.5)	23 (38.3)	0.305
Delirium, n (%), n = 93	9 (19.6)	13 (27.7)	0.358

*Early functional and cognitive outcome*			
mRS at 3 months, median (IQR)	1 (0–1)	2 (1–2)	**< 0.001**
Total cognitive Z-score at 6 months, median (IQR), n = 117	2.43 (−2.20–5.02)	−0.71 (−5.56–3.65)	0.024^a^

*Functional outcome at* 24 months			
mRS at 24 months, median (IQR)	0 (0–1)	1 (0–2)	**< 0.001**
Working full time, n (%)	37 (60.7)	33 (55)	0.529
Old age pension, n (%)	20 (32.8)	15 (25)	0.345
On sick leave or disability pension, n (%)	2 (3.3)	4 (6.7)	0.439

*SARS-CoV-2* Severe acute respiratory syndrome coronavirus 2; *IQR* interquartile range; *BMI* Body mass index; *LOS* length of stay; *mRS* modified Rankin Scale. Statistically significant p-values in bold.

a non-significant after FDR correction.

### 3.6 Functional outcome

At 24 months in all COVID-19 patient groups, functional outcome was good with a median mRS of 1 implying no disability despite some symptoms (Table 1). In pairwise comparisons, no significant differences existed between different COVID-19 severity groups (p = 0.724). Most of the patients were working full-time (ICU 53.4%, WARD 54.3%, HOME 71.4%) and more than one in four (28.9%) were on an old age pension; no significant differences existed between the severity groups (p = 0.252 and p = 0.11 respectively) (Table 1). Of those who were in working life before COVID-19, 76.7% (66/86) were working full-time and 11.6% (10/86) had retired meaning that the remaining 10 of 86 had not returned to full-time work; no differences between the acute disease severity groups existed (Table 1). One subject in both the ICU and WARD groups, along with two subjects in the HOME group reported working part-time because of COVID-19; one WARD subject was on sick leave because of COVID-19; two ICU subjects were on a disability pension because of COVID-19.

## 4 Discussion

In this 24-month longitudinal follow-up study of COVID-19 patients, approximately 30% of patients experienced clinically significant subjective cognitive symptoms, one-third reported fatigue, 27% reported at least mild anxiety symptoms, and around 10% at least moderate depressive or post-traumatic stress symptoms. No differences existed between acute disease severity groups. In the follow-up period from six to 24 months, patients who had clinically significant symptoms at six months showed improvement in symptoms of depression, anxiety, and post-traumatic stress, but not in subjective cognition or fatigue. However, the functional outcomes were positive.

In our study, 30% of patients experienced clinically significant subjective cognitive symptoms with no improvement over the follow-up period. Previous studies have reported similar results. In three studies with follow-up for 2–3 years, subjective cognitive symptoms in COVID-19 patients remained significant, at 20.8% for ICU-treated patients (Heesakkers et al., 2023), at 51% for hospitalised patients (Taquet et al., 2024), and at 15.9% for non-hospitalised patients (Fernandez-de-Las-Penas et al., 2022b). In a meta-analysis, two years post-COVID-19, subjective cognitive problems occurred in 27.6% (Fernandez-de-Las-Penas et al., 2024).

Our prevalence of clinically significant symptoms of 12.4% in depression and 27.3% in anxiety are in concordance with previous studies which have, however, reported very diverse prevalence rates. In our cohort, no significant differences existed between different acute disease severity groups. At two years post-COVID-19, studies have shown varying prevalence of depression and anxiety symptoms of 3.8–53.5% in hospitalised and non-hospitalised COVID-19 survivors (Guillen-Burgos et al., 2023; Huang et al., 2022; Li et al., 2022; Taquet et al., 2024; Zhang et al., 2023). In two meta-analyses, with patients of different acute disease severity, the proportion of patients with depression symptoms ranged from 6.6 to 18% and with anxiety symptoms from 9 to 13.4% (Fernandez-de-Las-Penas et al., 2024; Rahmati et al., 2023).

In our study, concerning depression and anxiety, signs of both deterioration and improvement existed: the frequency of clinically significant anxiety increased over the follow-up period, albeit non-significantly, but symptom scores in those with clinically significant symptoms at six months improved. In one study, symptom scores for depression and anxiety increased over a follow-up period from six months to 2–3 years, both because of the worsening of existing symptoms and the emergence of new symptoms (Taquet et al., 2024). A similar trend emerged for ICU-treated COVID-19 patients: mental symptoms, particularly anxiety, increased from one to two years (Heesakkers et al., 2023). However, opposite results exist: in a large follow-up study from six to 24 months, the frequency of anxiety and depressive symptoms decreased from 23 to 12% (Huang et al., 2022). A study defining recovery trajectories for mainly non-hospitalised COVID-19 patients showed that the majority of survivors (68.4%) recovered or continued to recover over the follow-up period from six to 24 months; however, some did not recover (4.4%), some worsened (5.2%) or had alternating (8.5%) trajectories (Ballouz et al., 2023). This alternating trajectory was evident also in our study where up to two-thirds of patients who exceeded some symptom cut-off at 24 months were below the cut-off at six months. Undoubtedly, the longer the follow-up period, the bigger is the role of confounding factors and, thus, any association with the index event becomes weaker.

For post-traumatic stress symptoms, two years after COVID-19, our prevalence of 9.9% is similar to two cohorts, hospitalised and ICU-treated, with a prevalence of 9–10.5% (Heesakkers et al., 2023; Li et al., 2022). In a large Chinese cohort, the proportion of COVID-19 survivors with PTSD symptoms was only 2% (Huang et al., 2022) whereas in a Colombian cohort, the prevalence of post-traumatic symptoms was 35% (Guillen-Burgos et al., 2023).

In our study, no improvement in fatigue symptoms over the follow-up occurred and the prevalence of clinically significant symptoms was relatively high at 35.5%. This is consistent with previous studies reporting fatigue as the most common post-COVID-19 symptom (Fernandez-de-Las-Penas et al., 2022b; Huang et al., 2022). Similarly, studies with two to three years of follow-up, including both hospitalised and non-hospitalised patients, have reported high fatigue prevalence of 31–62.3% in COVID-survivors (Fernandez-de-Las-Penas et al., 2022b; Huang et al., 2022; Kirchberger et al., 2023; Taquet et al., 2024). In a cohort of COVID-19 patients aged over 60, the prevalence of fatigue was lower at 7.7% (Zhang et al., 2023). In two meta-analyses, two years post-COVID-19, fatigue occurred in 27.4–28.0% (Fernandez-de-Las-Penas et al., 2024; Rahmati et al., 2023).

To summarise, our main findings were persistent subjective cognitive symptoms and fatigue affecting one in three COVID-19 survivors. We did not collect data about their inflammatory status, hence no conclusion about an inflammatory mechanism can be drawn. No differences between the severity groups existed so critical illness seems to be an unlikely explanation to the neuropsychiatric symptoms our patients suffered from. Psychosocial stress factors most likely played a role, because correlations existed between the different symptoms. Also, particularly for psychiatric symptoms, the symptom trajectories were alternating so that many with symptoms at 2 years were symptom-free at six months; thus factors unrelated to COVID-19 possibly had some effect.

In addition to COVID-19, long-term neuropsychiatric consequences occur after several conditions with differing pathophysiology in the acute phase. Sepsis survivors suffer from cognitive impairment, depressive and post-traumatic stress symptoms (Barichello et al., 2019), survivors after critical illness such as acute respiratory distress syndrome suffer from cognitive impairment, post-traumatic stress, anxiety, and depression (Davydow et al., 2008; Karnatovskaia et al., 2015; Sasannejad et al., 2019), whereas stroke and transient ischemic attack survivors suffer most often from depression and cognitive impairment, but also from anxiety (Chemerinski and Levine, 2006; El Husseini et al., 2023; Moran et al., 2014; Robinson, 1997); these symptoms are known to persist up to several years. However, comparison of our cohort with these patient groups is not straightforward: all of our patients were not critically ill or even hospitalised, and COVID-19 is not causing direct brain injury or functional disability comparable to stroke which was seen in our previous study where, six months after COVID-19, ischemic lesions and multiple cerebral microbleeds visible in brain magnetic resonance imaging were rare (Ollila et al., 2024).

We were able to identify only one factor, lower mRS at 3 months as a measure of functional outcome, associated with persistent symptoms at two years. Other studies with a follow-up of a minimum of two years have identified factors like impaired recovery at six months, history of a psychiatric or neurological condition, certain biocognitive profile in the acute phase, any comorbidity, age 65 years or older, history of fatigue, dyspnoea or problems in health-related quality of life, sociodemographic factors, acute phase disease severity, length of hospital stay, and mechanical ventilation as risk factors for persistent symptoms (Ballouz et al., 2023; Guillen-Burgos et al., 2023; Taquet et al., 2024). However, these results of risk factors have been inconsistent.

Nearly 90% of our cohort was either working full-time or on an old age pension with good functional outcome measured by the mRS. Comparison to other studies is fairly difficult because reported outcomes differ. In ICU-treated COVID-19 patients, over the follow-up from one to two years, despite persisting symptoms, work-related problems decreased from 66 to 32% (Heesakkers et al., 2023). At two years post-COVID-19, 89% of hospitalised patients had returned to their original work (Huang et al., 2022). Two to three years after COVID-19, 26.9% of patients reported occupational change, most often because of poor health; objective and subjective cognitive deficits, as well as fatigue, were associated with this change (Taquet et al., 2024). In a meta-analysis, two years post-COVID-19, 14.1% had not returned to work (Rahmati et al., 2023). This result is in accordance with our cohort, where, of those in working life before COVID-19, 11.6% had not returned to full-time work at two years.

Our study has limitations. First, our cohort was relatively small, single-centred, and predominantly of white ethnicity due to the language sensitivity of the 6-month neuropsychological testing. This introduced a selection bias. Second, we only gathered self-reported symptoms, but with formal questionnaires. Third, we collected our cohort during the first and second waves of COVID-19 when vaccines were unavailable; thus, our results are solely applicable to unvaccinated individuals infected with the original Wuhan variant. Fourth, we did not collect information about the patients' vaccination status at 24 months, or any new comorbidities diagnosed during the follow-up period, and re-infection rates were self-reported. All those factors might affect cognitive, psychiatric and fatigue outcomes. Our strengths include longitudinal follow-up from six to 24 months, inclusion of three different acute disease severity groups, and a fairly good response rate of 65%.

## 5 Conclusions

Our study adds to the growing evidence about the long-term effects of COVID-19 on subjective cognition, psychiatric, and fatigue symptoms as well as functional outcome. Our results showed that subjective cognitive symptoms and fatigue affected one in three patients, with no differences between acute severity groups, but being symptomatic in any domain had no clinically significant effect on employment status or functional disability. The median symptom scores in all domains were substantially below the cut-off limits implying that in general, our patients suffered from mild symptoms. On the other hand, patients with clinically significant symptoms of depression, anxiety or post-traumatic stress at six months, experienced improvement in symptom scores during the follow-up. Future studies are needed to add evidence of the effects of vaccinations and newer virus variants on long-term consequences.

## CRediT authorship contribution statement

**Henriikka Ollila:** Writing – review & editing, Writing – original draft, Visualization, Methodology, Investigation, Funding acquisition, Formal analysis, Conceptualization. **Marjaana Tiainen:** Writing – review & editing, Methodology, Investigation. **Riikka Pihlaja:** Writing – review & editing, Methodology, Investigation. **Sanna Koskinen:** Writing – review & editing, Investigation. **Annamari Tuulio-Henriksson:** Writing – review & editing, Investigation. **Viljami Salmela:** Writing – review & editing, Visualization, Investigation, Formal analysis. **Laura Hokkanen:** Writing – review & editing, Supervision, Methodology, Investigation, Formal analysis, Conceptualization. **Johanna Hästbacka:** Writing – review & editing, Supervision, Methodology, Investigation, Funding acquisition, Formal analysis, Conceptualization.

## Funding

H.O. has received funding from the 10.13039/501100007417Paulo Foundation and the 10.13039/100008723Finnish Medical Foundation (grant number 6417). J.H. has received Government Funding for University-level Research (TYH2021310) and from 10.13039/501100004785NordForsk. Open access funded by Helsinki University Library. The funders had no role in the study design, data collection and analysis, decision to publish, or preparation of the manuscript.

## Declaration of competing interest

The authors declare the following financial interests/personal relationships which may be considered as potential competing interests: Henriikka Ollila reports financial support was provided by 10.13039/501100007417Paulo Foundation. Henriikka Ollila reports financial support was provided by 10.13039/100008723Finnish Medical Foundation. Johanna Hastbacka reports financial support was provided by Government Funding for University level Research. Johanna Hastbacka reports financial support was provided by Nordforsk. If there are other authors, they declare that they have no known competing financial interests or personal relationships that could have appeared to influence the work reported in this paper.

## Data Availability

Upon reasonable request from the corresponding author, some anonymous data can be shared. The data are not publicly available due to privacy restrictions.
