# Supplementary material for: Subjective cognitive, psychiatric, and fatigue symptoms two years after COVID-19: A prospective longitudinal cohort study
Source: Brain Behav Immun Health. 2025 Mar 18;45:100980. doi: 10.1016/j.bbih.2025.100980 (PMC11978368; doi:10.1016/j.bbih.2025.100980)
Supplement: Multimedia component 1 [file mmc1.pdf]

Subjective cognitive, psychiatric, and fatigue symptoms two years after COVID-19: a prospective longitudinal cohort study

Henriikka Ollila[a,*], Marjaana Tiainen[b], Riikka Pihlaja[c,d], Sanna Koskinen[c], Annamari Tuulio-Henriksson[c], Viljami Salmela[c], Laura Hokkanen[c], Johanna Hästbacka[e,f]

[a] Perioperative and Intensive Care, Helsinki University Hospital and University of Helsinki, Helsinki, Finland
[b] Department of Neurology, Helsinki University Hospital and University of Helsinki, Helsinki, Finland
[c] Department of Psychology, Faculty of Medicine, University of Helsinki, Helsinki, Finland
[d] Division of Neuropsychology, HUS Neurocenter, Helsinki University Hospital and University of Helsinki, Helsinki, Finland
[e] Department of Intensive Care, Tampere University Hospital, Wellbeing Services County of Pirkanmaa, Tampere, Finland
[f] Tampere University, Faculty of Medicine and Health Technology, Tampere, Finland
[*] Corresponding author, email address henriikka.ollila@helsinki.fi

Table S1. Number and proportions of patients who exceeded a cut-off level at 24 months divided in two groups based on whether they exceeded the cut-off at six months. McNemar test for paired samples was utilised to compare the proportions above and below the cut-off level at six and 24 months.

	Below cut-off at six months	Above cut-off at six months	Total, n = 121	p, McNemar test
ABNAS at 24 months				
Below cut-off, n (%)	67 (79.8)	17 (20.2)	84 (69.4)	0.353
Above cut-off, n (%)	12 (32.4)	25 (67.6)	37 (30.6)	
PHQ-9 at 24 months				
Below cut-off, n (%)	97 (91.5)	9 (8.5)	106 (87.6)	1.00
Above cut-off, n (%)	9 (60)	6 (40)	15 (12.4)	
GAD-7 at 24 months				
Below cut-off, n (%)	79 (89.8)	9 (10.2)	88 (72.7)	0.059
Above cut-off, n (%)	19 (57.6)	14 (42.4)	33 (27.3)	
IES-6 at 24 months				
Below cut-off, n (%)	92 (84.4)	17 (15.6)	109 (90.1)	0.072
Above cut-off, n (%)	8 (66.7)	4 (33.3)	12 (9.9)	
MFI at 24 months				
Below cut-off, n (%)	63 (79.7)	15 (19.2)	78 (64.5)	0.857
Above cut-off, n (%)	16 (37.2)	27 (62.8)	43 (35.5)	

ABNAS AB Neuropsychological Assessment Schedule; *PHQ9* Patient Health Questionnaire 9; *GAD7* Generalised Anxiety Disorder 7; *IES-6* Impact of Event Scale 6; *MFI* Multidimensional Fatigue Inventory

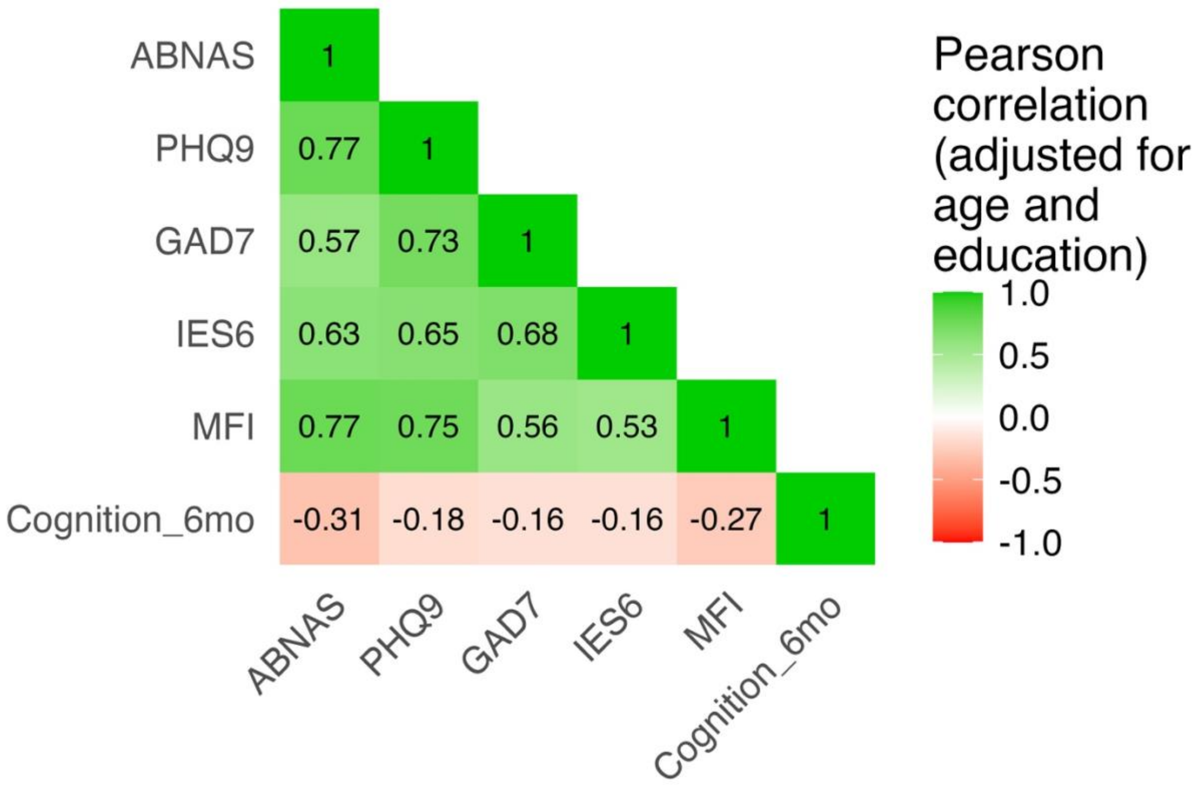

Figure S1. Analysis of Pearson correlations for ABNAS, PHQ-9, GAD-7, IES-6, and MFI scores at 24 months presented as a heat map. Results are adjusted for age and educational years. The total cognitive Z-score at 6 months (=Cognition_6mo) was included in the analysis. Correlations > 0.2 are statistically significant ($p < 0.05$).

Table S2. Symptom score means at six and 24 months after COVID-19 in patients below and above cut-off levels at six months.

	All COVID-patients Score, mean (SEM)		p	ICU Score, mean (SEM)		p	WARD Score, mean (SEM)		p	HOME Score, mean (SEM)		p
	At 6 months	At 24 months		At 6 months	At 24 months		At 6 months	At 24 months		At 6 months	At 24 months	
ABNAS at 6 months												
Below cut-off, n = 79	6.47 (0.84)	8.11 (1.26)	0.494	6.69 (1.16)	9.27 (1.73)	0.902	5.14 (1.60)	7.43 (2.38)	0.996	7.57 (1.61)	7.64 (2.41)	1.0
Above cut-off, n = 42	24.43 (1.19)	21.63 (1.78)	0.330	23.83 (1.64)	20.74 (2.45)	0.968	24.76 (1.84)	22.12 (2.75)	0.996	24.68 (2.64)	22.02 (3.95)	1.0
PHQ-9 at 6 months												
Below cut-off, n = 106	3.15 (0.26)	3.34 (0.37)	0.960	3.17 (0.36)	3.32 (0.52)	1.0	3.22 (0.48)	3.28 (0.69)	1.0	3.07 (0.52)	3.43 (0.75)	1.0
Above cut-off, n = 15	13.03 (0.71)	8.85 (1.02)	**< 0.001**	12.92 (1.15)	7.50 (1.65)	0.775	13.29 (0.96)	9.57 (1.38)	0.256	12.89 (1.49)	9.47 (2.14)	0.915
GAD-7 at 6 months												
Below cut-off, n = 98	1.48 (0.17)	2.02 (0.29)	0.203	1.64 (0.24)	2.14 (0.41)	0.978	1.65 (0.31)	2.13 (0.52)	0.998	1.14 (0.34)	1.78 (0.59)	0.991
Above cut-off, n = 23	7.52 (0.36)	5.0 (0.62)	**< 0.001**	6.60 (0.49)	4.28 (0.83)	0.130	8.95 (0.61)	5.44 (1.04)	**0.024**	7.02 (0.74)	5.29 (1.27)	0.950
IES-6 at 6 months												
Below cut-off, n = 100	0.60 (0.05)	0.46 (0.06)	0.135	0.58 (0.07)	0.59 (0.09)	1.0	0.67 (0.08)	0.51 (0.11)	0.939	0.54 (0.10)	0.28 (0.13)	0.660
Above cut-off, n= 21	2.24 (0.11)	1.06 (0.15)	**< 0.001**	2.26 (0.13)	0.79 (0.17)	**< 0.001**	2.34 (0.20)	1.36 (0.27)	**0.025**	2.12 (0.23)	1.03 (0.30)	**0.030**
MFI at 6 months												
Below cut-off, n = 79	40.5 (1.37)	43.6 (1.79)	0.302	43.5 (1.77)	44.3 (2.32)	1.0	38.7 (2.45)	42.9 (3.22)	0.967	39.5 (2.82)	43.6 (3.7)	0.992
Above cut-off, n = 42	70.5 (1.76)	65.2 (2.31)	0.089	70.9 (2.75)	69.3 (3.61)	1.0	70.7 (3.01)	63.6 (3.94)	0.946	69.8 (3.42)	62.7 (4.49)	0.889

SEM standard error of mean; *ABNAS* AB Neuropsychological Assessment Schedule; *PHQ9* Patient Health Questionnaire 9; *GAD7* Generalised Anxiety Disorder 7; *IES-6* Impact of Event Scale 6; *MFI* Multidimensional Fatigue Inventory. Statistically significant p-values in bold.
